# Genomic selection models substantially improve the accuracy of genetic merit predictions for fillet yield and body weight in rainbow trout using a multi-trait model and multi-generation progeny testing

**DOI:** 10.1186/s12711-023-00782-6

**Published:** 2023-02-09

**Authors:** Andre Garcia, Shogo Tsuruta, Guangtu Gao, Yniv Palti, Daniela Lourenco, Tim Leeds

**Affiliations:** 1 Department of Animal and Dairy Science, University of Georgia, Athens, GA 30602 USA; 2 National Center for Cool and Cold Water Aquaculture, Agricultural Research Service, United States Department of Agriculture, Kearneysville, WV 25430 USA

## Abstract

**Background:**

In aquaculture, the proportion of edible meat (FY = fillet yield) is of major economic importance, and breeding animals of superior genetic merit for this trait can improve efficiency and profitability. Achieving genetic gains for fillet yield is possible using a pedigree-based best linear unbiased prediction (PBLUP) model with direct and indirect selection. To investigate the feasibility of using genomic selection (GS) to improve FY and body weight (BW) in rainbow trout, the prediction accuracy of GS models was compared to that of PBLUP. In addition, a genome-wide association study (GWAS) was conducted to identify quantitative trait loci (QTL) for the traits. All analyses were performed using a two-trait model with FY and BW, and variance components, heritability, and genetic correlations were estimated without genomic information. The data used included 14,165 fish in the pedigree, of which 2742 and 12,890 had FY and BW phenotypic records, respectively, and 2484 had genotypes from the 57K single nucleotide polymorphism (SNP) array.

**Results:**

The heritabilities were moderate, at 0.41 and 0.33 for FY and BW, respectively. Both traits were lowly but positively correlated (genetic correlation; r = 0.24), which suggests potential favourable correlated genetic gains. GS models increased prediction accuracy compared to PBLUP by up to 50% for FY and 44% for BW. Evaluations were found to be biased when validation was performed on future performances but not when it was performed on future genomic estimated breeding values.

**Conclusions:**

The low but positive genetic correlation between fillet yield and body weight indicates that some improvement in fillet yield may be achieved through indirect selection for body weight. Genomic information increases the prediction accuracy of breeding values and is an important tool to accelerate genetic progress for fillet yield and growth in the current rainbow trout population. No significant QTL were found for either trait, indicating that both traits are polygenic, and that marker-assisted selection will not be helpful to improve these traits in this population.

## Background

Fillet yield is the ratio between the edible portion (meat) and the whole weight of the fish at harvest, and this trait is of primary economic importance in aquaculture. The price paid when fish are sold as fillet can be much higher than the price of whole fish; therefore, small changes in fillet yield can result in a significant economic impact on the production chain [1, 2]. In fact, a study by Sae-Lim et al. [3] ranked fillet yield among the six most important traits for genetic improvement in rainbow trout breeding programs. Although the benefits of improving fillet yield are clear, implementing a selection program for this trait is challenging for several reasons. First, phenotypes cannot be recorded on selection candidates; second, even when phenotyping is available, it is usually at a costly and laborious process; and third, fillet yield is a ratio trait, which can be difficult to model. In spite of these challenges, studies using simulation and real data showed that improving fillet yield is possible by using direct or indirect selection and adjusting trait definitions for better modelling [4–6].

The recent development of high-density single nucleotide polymorphism (SNP) panels has added an extra resource in the toolbox of breeding programs. It allows for the prediction of more accurate breeding values and ultimately leads to higher genetic gains [7]. The use of genomic information has been widely incorporated in livestock populations and more recently in aquaculture breeding programs for several species, for instance, salmon, trout, catfish, and tilapia [8–12]. In rainbow trout populations, genomic information has been used across breeding populations to study and evaluate several traits such as growth [13], disease resistance [14–16], and carcass [17]. All these studies reported the benefits of using genomic information in aquaculture breeding programs. Furthermore, genomic information is especially beneficial for traits that cannot be measured on selection candidates or have a low heritability, such as resistance to disease and carcass traits [7, 18].

Gonzalez-Pena et al. [19] performed genomic analyses for fillet yield, carcass, and body weight, using single-trait models in a rainbow trout population from the National Center for Cool and Cold Water Aquaculture (NCCCWA) and found that the use of genomic information was beneficial to explore within-family variation and to obtain faster genetic gains for these traits. Our overall goal in the current study was to build on those results by using additional data and a multiple trait approach to investigate the prediction accuracy and bias of genomic predictions and further understand the usefulness of genomic selection for fillet yield in rainbow trout. Our specific objectives were to (1) evaluate the prediction accuracy of traditional and genomic evaluations with different genomic models, and (2) use a two-trait model with fillet yield and body weight, to estimate variance components, heritability, and genetic correlations.

## Methods

### Data and resource population

Data were collected by the experimental breeding program of the USDA National Center for Cool and Cold Water Aquaculture (NCCCWA; Leetown, WV). Phenotypes were recorded for fillet yield (FY; N = 2642) from 2010 to 2018 and for 13-month BW, (hereafter referred to as body weight) (BW; N = 12,890) from 2004 to 2016 (Table 1). For the 2018 hatch year, BW data were not available because animals were only measured later at harvest time (i.e., 14.5 vs. 13 months). The total number of animals with pedigree records was 14,165, from which 2484 were genotyped using the 57K SNP Axiom trout genotyping array [20]. After filtering for quality control, 34,251 informative SNPs were used in the data analysis.

**Table 1:** Summary statistics for body weight and fillet yield

Trait^a^	Number of records	Mean	Min	Max	SD
Body weight	12,890	1043	42.9	2565	346
Fillet yield	2642	50.9	34.8	59.5	2.97

^a^Body weight was measured in grams and fillet yield in %

A fully-pedigreed line selected for growth performance served as the founder population for this study. A target of five fish from each third- (n families = 98; hatch year 2010), fourth- (n families = 99; hatch year 2012), and fifth-generation family (n = 102; hatch year 2014) of the ‘Select’ line described in Leeds et al. [21] were phenotyped for fillet yield. Briefly, fish were sampled from each family to represent within-family variation in growth performance at approximately 13 months post-hatch. Each family had approximately 15 fish eligible for sampling. Sampling was conducted by sorting fish within each family by descending body weight and identifying every second or third fish for sampling, with the exception that fish with a body weight that was more or less than 3 standard deviations from the family mean were excluded from sampling. Fish were then assigned to one of the five harvest groups (i.e., one harvest group per week for each of 5 consecutive weeks) in each generation. The aim was to have one fish per family represented in each harvest group, and fish were assigned to harvest groups in descending order of body weight such that the heaviest fish were harvested in the first harvest group and the lightest fish were harvested in the last harvest group. At approximately 14.5 months post-hatch, fish were euthanized using a lethal dose of tricaine methanesulfonate (Tricaine-S, Western Chemical, Ferndale, WA), eviscerated, and stored overnight on ice. Carcasses were hand-filleted the following day by a trained technician. Filleting was conducted for families hatched in 2010 and 2012 at West Virginia University (Davis College of Agriculture, Forestry and Consumer Sciences, Morgantown, WV); then, filleting was conducted at the NCCCWA. The skin was removed from all fillets from fish of families hatched in 2010 and 2012 and it was left on the fillet from fish of families hatched after 2012. All carcasses of each generation were filleted by a single technician. Fillet yield was calculated as total fillet weight/BW at harvest.

The breeding objective of the program changed from growth performance to fillet yield in 2014, thus the three generations of fillet yield data were used to estimate family-based breeding values for families hatched in 2014. Based on those family breeding values, a divergent selection was applied to develop contemporary high fillet yield (ARS-FY-H) and low fillet yield (ARS-FY-L) lines, starting from 2016. Breeding values were estimated for each generation using a three-trait animal model that included fillet yield, 10-month BW, and thermal growth coefficient (defined as function of growth in a growing period and the average water temperature in that period) [21], using MTDFREML [22]. The model for fillet yield included fixed effects for hatch year, harvest group nested within hatch year, and harvest BW (linear covariate) and random animal, full-sib family, and rearing tank effects. The latter two traits were included to account for the prior selection that the population was subjected to, and the effects included in the model are given in Leeds et al. [21]. Selection and mating decisions were made in each generation to maximize genetic gain while constraining inbreeding accumulation to ≤ 1% per generation as described in Leeds et al. [21]. The goal was to produce 100 ARS-FY-H and 23 ARS-FY-L families in each generation, although the actual number in each year may differ due to practical and logistical reasons. Whereas upward selection was practiced in each generation for the ARS-FY-H line, the ARS-FY-L line was subjected to only one generation of downward selection and, after that, mated at random to maintain genetic diversity within the line. To produce the first (hatch year 2016) and second (hatch year 2018) generations of the ARS-FY-H nucleus families, sires and dams were selected based on family FY breeding values from 32 (hatch year 2014) and 51 (hatch year 2016) parental families, resulting in weighted phenotypic selection differentials for FY of + 1.533 and + 1.324 percentage points, respectively. Likewise, to produce first-generation ARS-FY-L nucleus families, sires and dams were selected based on family FY breeding values (downward selection) from 31 parental families (hatch year 2014). To produce second-generation ARS-FY-L nucleus families, sires and dams were sampled from 22 parental families (hatch year 2016). The resulting weighted phenotypic selection differentials for FY in the ARS-FY-L line were − 1.377 and − 0.088 percentage points in the first and second generations, respectively. Overall, grow-out, tagging, and phenotyping of families hatched in 2016 and 2018 were consistent with those described above for families hatched in 2014 and before.

### Model and estimation of variance components

The following two-trait animal model was used in the analyses:

$${\mathbf{y}}_{\text{t}}= {\mathbf{X}}{\mathbf{b}}_{\mathrm{t}}\text{ + }{\mathbf{Z}}_{\boldsymbol{{1}}}{\mathbf{u}}_{\mathrm{t}}\text{ + }{\mathbf{Z}}_{2}{\mathbf{f}}_{\mathrm{t}}\text{ + }{\mathbf{e}},$$

where ${\mathbf{y}}_{\text{t}}$ is the vector of phenotypes and $\mathrm{t}$ refers to each trait (BW and FY); ${\mathbf{b}}_{\mathrm{t}}$ is the vector of fixed effects. The model for FY included harvest age as a covariable, line (ARS-FY-H and ARS-FY-L), and the interaction between harvest year and slaughter group. The model for BW included age as a covariable, line and hatch year; ${\mathbf{u}}_{\mathrm{t}}$ and ${\mathbf{f}}_{\mathrm{t}}$ are the vectors of the additive genetic and family random effects, and **e** is the vector of residuals. The ${\mathbf{X}}$, ${\mathbf{Z}}_{1}$, and ${\mathbf{Z}}_{2}$ are the incidence matrices for the effects in ${\mathbf{b}}_{\mathrm{t}}$, ${\mathbf{u}}_{\mathrm{t}}$ and ${\mathbf{f}}_{\mathrm{t}}$, respectively. The line effect was included in the model because, over the years, different selection criteria were used to select animals, creating different lines within the same population.

The traditional evaluation was performed using pedigree-based best linear unbiased prediction (PBLUP) and genomic evaluations were performed using single step genomic BLUP (ssGBLUP) [23, 24]. In ssGBLUP, the inverse of the pedigree relationship matrix (${\mathbf{A}}^{-{\boldsymbol{1}}}$) is replaced by the inverse of the relationship matrix combining pedigree and genomic relationships (${\mathbf{H}}^{-{\boldsymbol{1}}}$), as in Aguilar et al. [23]:

$${\mathbf{H}}^{-{\boldsymbol{1}}} = {\mathbf{A}}^{-{1}}\text{ + }\left[\begin{array}{cc}{\boldsymbol{0}}& {\boldsymbol{0}}\\ {\boldsymbol{0}}& {\mathbf{G}}^{-{\boldsymbol{1}}}-{\mathbf{A}}_{\boldsymbol{22}}^{-\boldsymbol{1}}\end{array}\right],$$

where ${\mathbf{G}}^{-{\boldsymbol{1}}}$ is the inverse of the genomic relationship matrix and ${\mathbf{A}}_{\boldsymbol{22}}^{-1}$ is the inverse of the pedigree relationship matrix for genotyped animals. The genomic relationship matrix ($\mathbf{G}$) was constructed as in VanRaden [25]:

$$\mathbf{G} = \frac{{\mathbf{Z}}{\mathbf{D}}{\mathbf{Z}}^{\mathbf{^{\prime}}}}{{2}\sum {\text{p}}_{\text{i}}\text{(1-}{\text{p}}_{\text{i}}\text{)}},$$

where $\mathbf{Z}$ is the matrix of genotypes centered by allele frequencies from the base population, and ${\text{p}}_{\text{i}}$ is the allele frequency of the $\mathrm{i}$-th SNP, which was also from the base population. These allele frequencies were computed based on Gengler et al. [26]. If base allele frequencies can be accurately computed, there is no need to use methods to ensure compatibility between ${\mathbf{G}}$ and ${\mathbf{A}}_{\boldsymbol{22}}$ [27]. In the unweighted ssGBLUP, $\mathbf{D}$ a matrix of weights for SNP is equal to $\mathbf{I}$, and all markers are assumed to explain the same proportion of genetic variance.

Variance components, heritability, and genetic correlations were estimated without genomic information using the model described above, under PBLUP, implemented in the AIREMLF90 software [28].

### Validation

We investigated the impact of including genomic information in FY and BW evaluations using two datasets to perform a mid-parent validation for FY and two forward validation methods based on young, genotyped animals for FY and BW. The datasets and validation strategies are described below.

### Mid-parent validation

This practical validation strategy that directly estimates genetic improvement across generations is used in aquaculture when progeny phenotypes are available and can be used as a benchmark to compare with parent averages (PA), which are equal to the average genomic estimated breeding values (GEBV) of parents [19, 29]. In our study, parental genotypes were available for 96 of the 99 ARS-FY-H nucleus families hatched in 2018. After removing half sib families, phenotypes for the fish hatched in 2018 from 73 full-sib families were kept for validation. Phenotypes of five animals from each family were averaged and compared to the parental average breeding value from PBLUP and ssGBLUP. In addition, instead of raw phenotypes, phenotypes adjusted for fixed effects were used as a benchmark.

For the 2018 hatch year, BW records were not available because the animals were only measured later at harvest. Since harvest weight is measured later than BW (i.e., 14.5 vs. 13 months), we could not perform the mid-parent validation for BW.

The correlation of PA and average phenotypes over all the 73 families is reported as a measure of prediction accuracy, and the coefficient of the regression of PA on (G)EBV is reported as a measure of dispersion bias of the breeding values.

### Forward validation

With the advent of genomic selection, and since the main goal of genetic evaluations is to predict future performance, using young, genotyped animals for validation became the standard method to validate predictions [30]. In our study, we chose 562 genotyped animals from the 2016 hatch year, with records for both BW and FY as validation animals. The validation on young animals was performed using two methods, one was based on adjusted phenotypes and the other was the linear regression method (LR method) as described by Legarra and Reverter [31].

For the validation based on adjusted phenotypes, the phenotypes were adjusted for the fixed effects calculated with PBLUP with the whole data, i.e. $({\mathbf{y}}^{\mathbf{*}}=\mathrm{y}-{\mathbf{X}}{\text{b}}- {\mathbf{Z}}_{2}{\text{f}})$. Then, traditional and genomic evaluations were computed with partial data by omitting the data for animals born in hatch year 2016 and onwards, and finally, accuracy, bias, and dispersion were calculated as follows:

$$\mathrm{Accuracy}=\frac{\mathrm{cor}({\mathrm{y}}^{*}, \widehat{\mathrm{u}})}{\sqrt{{\mathrm{h}}^{2}}},$$

$${\mathrm{y}}^{*}= {\mathrm{b}}_{0}+{\mathrm{b}}_{1}\widehat{\mathrm{u}},$$

where $\widehat{\mathbf{u}}$ is a vector of (G)EBV, and ${\mathrm{b}}_{0}$ and ${\mathrm{b}}_{1}$ are the intercept and the regression coefficients of adjusted phenotypes on (G)EBV, respectively. The ${\mathrm{b}}_{0}$ and ${\mathrm{b}}_{1}$ coefficients are measures of bias and dispersion of (G)EBV, respectively. The predictive ability, i.e., $\mathrm{cor}({\mathbf{y}}^{*}, \widehat{\mathbf{u}})$, was divided by the square root of the heritability to make it comparable across traits and validation strategies, as the prediction accuracy of the models.

For the LR validation, traditional and genomic evaluations were run with whole (w) and partial (p) datasets. In the partial dataset, phenotypes for the validation animals are omitted. In this validation, we can evaluate the impact of including new data into subsequent evaluations and we can investigate the value of adding genomic data.

Once evaluations were run, the following four statistics described by Legarra and Reverter [31]. were computed to evaluate the models:

- (1) $$\mathrm{Accuracy }= \sqrt{\frac{\mathrm{cov}({\widehat{\mathrm{u}}}_{\mathrm{w}},{\widehat{\mathrm{u}}}_{\mathrm{p}})}{(1-\overline{\mathrm{F} }){\widehat{\upsigma }}_{\mathrm{u}}^{2}},}$$

where $\overline{\mathrm{F} }$ is the average inbreeding for the validation animals and ${\widehat{\upsigma }}_{\mathrm{u}}^{2}$ is the additive genetic variance.

- (2) $$\mathrm{Bias }= {\overline{\widehat{\mathrm{u}}} }_{\mathrm{p}}- {\overline{\widehat{\mathrm{u}}} }_{\mathrm{w}},$$

where ${\overline{\widehat{\mathrm{u}}} }_{\mathrm{p}}$ and ${\overline{\widehat{\mathrm{u}}} }_{\mathrm{w}}$ are the average breeding values for the validation animals, which are computed based on partial and whole data, respectively. The bias has an expected value of 0 if the evaluations are unbiased.

- (3) $$\mathrm{Slope}= {\mathrm{b}}_{\mathrm{w},\mathrm{p}} = \frac{\mathrm{cov}({\widehat{\mathrm{u}}}_{\mathrm{w}},{\widehat{\mathrm{u}}}_{\mathrm{p}})}{\mathrm{var}({\widehat{\mathrm{u}}}_{\mathrm{p}})}.$$

The slope of the regression of ${\widehat{\mathbf{u}}}_{\mathrm{w}}$ on ${\widehat{\mathbf{u}}}_{\mathrm{p}}$ can be an indicator of dispersion of (G)EBV, and ideally this slope would be 1 or close to 1.

- (4) $$\mathrm{Consistency}=\mathrm{cor}\left({\widehat{\mathrm{u}}}_{\mathrm{w}},{\widehat{\mathrm{u}}}_{\mathrm{p}}\right).$$

The higher this correlation is, the more consistent two subsequent evaluations are when new data are added.

All four statistics were computed for the 2016 hatch year validation animals (N = 562), in both the traditional and genomic evaluations. The number of genotyped animals with phenotypes in the training population for the mid-parent validation was 1929 and for the validation on young animals it was 1366.

### Weighted ssGBLUP (WssGBLUP) and genome-wide association study (GWAS)

From the ssGBLUP evaluation, GEBV can be backsolved into SNP effects which can help uncover the genetic architecture of a trait. In our study, SNP effects were calculated as in Wang et al. [32]:

$$\widehat{\mathbf{a}}= \lambda \mathbf{DZ^{\prime}}{\mathbf{G}}^{\boldsymbol{-1}}\widehat{\mathbf{u}},$$

where $\widehat{\mathbf{u}}$ is a vector of (G)EBV, $\widehat{\mathbf{a}}$ is a vector of SNP effects and the ${\mathbf{D}}$, ${\mathbf{Z}}$, and ${\mathbf{G}}^{-1}$ matrices are as previously defined.

Once SNP effects were calculated, the proportion of additive variance explained by windows of 20 adjacent SNP was calculated. The order of SNPs on the rainbow trout genome chromosomes was determined based on their position in the GenBank Assembly Accession GCA_002163495.1 [33]. In addition, p-values for marker effects were obtained using the procedure presented by Aguilar et al. [34] as follows:

$${pvalue}_{i}=2\left(1- \Phi \left(\left|\frac{{\widehat{a}}_{i}}{\mathrm{SD}\left({\widehat{a}}_{i}\right)}\right|\right)\right),$$

where $\Phi$ is the cumulative standard normal function. SNPs were declared significantly associated with FY or BW at a 5.8 threshold (corrected by Bonferroni) on the − log10 scale.

When significantly associated important SNPs are identified, differential weights can be attributed to them in the genomic evaluation for potential increases in accuracy. Under the ssGBLUP framework, this can be done by using WssGBLUP over a few iterations, i.e., three to five, to optimize the weights applied in the construction of $\mathbf{G}$ [32] by maximizing the accuracy of predictions. In our study, we applied the nonlinear A weights in the WssGBLUP model, as described by VanRaden [25] and Legarra et al. [35], as follows:

$${\mathrm{d}}_{\mathrm{i}}={\mathrm{CT}}^{\frac{|{\widehat{\mathrm{a}}}_{\mathrm{i}}|}{\mathrm{sd}(\widehat{\mathrm{a}})}-2},$$

where $\mathrm{CT}$ is a constant that determines the departure of SNP effects from normality; $|{\widehat{\mathrm{a}}}_{\mathrm{i}}|$ is the absolute value of the SNP effect $\mathrm{i}$, and $\mathrm{sd}\left(\widehat{\mathbf{a}}\right)$ is the standard deviation of the vector of SNP effects. Because $\mathrm{CT}$ is empirically derived, three values were tested in this population: 1.025, 1.125 and 1.25 to determine the value that led to the best prediction accuracy and the least bias in the evaluation.

The two-trait model was used, and SNP effects and weights for $\mathbf{G}$ were calculated one trait at a time, and the validation based on adjusted phenotypes (described above) was applied across five iterations of WssGBLUP. All the analyses were performed using software from the BLUPF90 family of programs [28].

## Results and discussion

### Estimates of variance components, heritabilities, and genetic correlations

The estimates of variance components, heritabilities, and genetic correlations for FY and BW are in Table 2. The heritability estimate for FY was 0.41, which is slightly higher than previously reported estimates that ranged from 0.30 to 0.35 [4, 19, 36], and similar to the heritability estimate of residual fillet weight (0.38) that Vandeputte et al. [6] proposed as an alternative trait to improve fillet yield. The heritability estimate for BW (0.33) was similar to the estimates found in the literature that range from 0.26 to 0.37 [4, 21].

**Table 2:** Estimates of variance components for fillet yield and body weight

	Fillet yield (SE)	Body weight (SE)
${\upsigma }_{\mathrm{u}}^{2}$	1.99 (0.42)	17,648 (2526)
${\upsigma }_{\mathrm{f}}^{2}$	0.24 (0.12)	5,679 (733)
${\upsigma }_{\mathrm{e}}^{2}$	2.60 (0.23)	29,913 (1319)
${\upsigma }_{\mathrm{p}}^{2}$	4.82 (0.18)	53,239 (1187)
${\mathrm{h}}^{2}$	0.41 (0.07)	0.33 (0.04)
${\mathrm{f}}^{2}$	0.05 (0.03)	0.11 (0.01)
r_g_	0.24 (0.14)	

${\upsigma }_{\mathrm{u}}^{2}$: additive genetic variance; ${\upsigma }_{\mathrm{f}}^{2}$: variance of the family effect; ${\upsigma }_{\mathrm{e}}^{2}$: residual variance; ${\upsigma }_{\mathrm{p}}^{2}$: phenotypic variance; ${\mathrm{h}}^{2}$: heritability; ${\mathrm{f}}^{2}$: proportion of variance explained by the family effect; r_g_: genetic correlation

Estimating the genetic correlation between FY and BW is important to understand the relationship and potential impacts of selection for either of the traits. The correlations reported in the literature range from very low (0.04) to moderately positive (0.22) [4, 36]. In our study, the genetic correlation between FY and BW was moderate and positive (0.24), indicating that selection for BW could result in indirect gains for FY. It is important to note that different data recording practices such as measuring BW and FY at constant age or constant harvest weight, and the modeling strategy adopted, can impact the estimates of genetic parameters, heritabilities and genetic correlations, and also impact the relationship between the traits, thus affecting potential correlated response to selection [36, 37].

### Prediction accuracy

Overall, the results of the three validation strategies used in our study agreed and the ssGBLUP evaluation always outperformed the traditional PBLUP. The results for the validation are in Tables 3, 4, and 5 for the mid-parent, young animals (adjusted phenotypes), and LR validations, respectively. For FY, the mid-parent validation was used to investigate the accuracy of predicting the realized phenotypes of the young animals from hatch year 2018. The gains in prediction accuracy with the genomic evaluation over the PBLUP evaluation reached 50% when using raw phenotypes (from 0.16 to 0.24), and 30% when using adjusted phenotypes (from 0.20 to 0.26). The percentage of gain when using adjusted phenotypes was in line with the other validation methods. For instance, using the validation on young animals from the 2016 hatch year, the gain in prediction accuracy with ssGBLUP was 27% (from 0.49 to 0.62) and with the LR method the gain was 37% (from 0.49 to 0.67). Often the main goal is to predict the genetic merit of the animals, and therefore, using the phenotypes adjusted by the fixed effects is a more appropriate benchmark [30].

**Table 3:** Results for the validation on mid-parent (G)EBV and average progeny phenotypes for fillet yield

Model	N	Fillet yield					
		Raw phenotypes			Adjusted phenotypes		
		cor	b0	b1	cor	b0	b1
BLUP	73	0.16	52.72	0.59	0.20	0.85	0.63
ssGBLUP	73	0.24	52.61	0.55	0.26	0.91	0.52

Body weight data (BW) were not collected from the year-class 2018 progeny that were phenotyped for FY

N: number of families in the validation sample

cor: correlation between average progeny phenotypes and (GEBV); b0: bias; b1: inflation

**Table 4:** Results for the validation with adjusted phenotypes

Model	N	Fillet yield			Body weight		
		Accuracy	b0	b1	Accuracy	b0	b1
BLUP	562	0.49	0.05	1.04	0.35	39.36	0.97
ssGBLUP	562	0.62	0.33	0.96	0.39	242.13	0.74

N: number of progeny with phenotype data for both traits in the validation panel (year-class of 2016)

Accuracy: [correlation between adjusted phenotypes and (GEBV)]/sqrt(h^2^); b0: bias; b1: inflation

**Table 5:** Results for the LR validation

Model	N	Fillet yield				Body weight			
		Accuracy	Bias	Slope	Consistency	Accuracy	Bias	Slope	Consistency
BLUP	562	0.49	− 0.06	1.03	0.63	0.32	− 7.88	0.92	0.52
ssGBLUP	562	0.67	− 0.09	0.99	0.79	0.46	− 12.61	0.90	0.68

N: number of progeny with phenotype

Because BW phenotypes were not available for fish in hatch year 2018, the animals born in 2016 were used in the validation. For this trait, the gains in accuracy with ssGBLUP were higher with the LR validation (44%) than with adjusted phenotypes (11%). Although genomic evaluation outperformed traditional PBLUP for both traits in both validations, this discrepancy in the magnitude of the gains could be due to stronger selection on BW, which may not be as well accounted for by the LR validation. In LR, the denominator of the accuracy formula is used to consider selection; however, the formula we used is an approximation to the main formula [31]. The denominator of the main formula requires the additive genetic variance in the validation set, which may be difficult to compute [38].

Similar gains in prediction accuracy have been reported for a variety of traits in aquaculture species, for instance, Atlantic salmon [8], rainbow trout [15, 16, 19], tilapia [11, 12], and channel catfish [10]. One example for which the reported gain in prediction accuracy was substantially greater (100% improvement over PBLUP) was for resistance to bacterial cold-water disease (BCWD) in rainbow trout [9]. We believe that, in that example, the presence of two to three major QTL for BCWD in this rainbow trout population [9] has been a major contributor to the enhanced improvement in the estimated accuracy of the genomic prediction compared to PBLUP prediction. These and many other studies demonstrate the benefit of incorporating genomic information into routine evaluations for aquaculture breeding programs. In addition, these benefits are further highlighted for traits with a low heritability or that cannot be measured on selection candidates, such as fillet yield and disease resistance. In addition, a recent simulation study predicted improvement in genetic merit prediction accuracy for carcass yield in rainbow trout by using genomic selection coupled with selection for an indirect morphological indicator [39].

Our study provides the first estimates of validation accuracy from genomic evaluation models for fillet yield in aquaculture using mid-parent validation [19, 29]. This is important because the ultimate practical test for selective breeding is the net genetic gain and actual improvement in performance in the next generation of the breeding program.

### Bias and dispersion of breeding values

Bias and inflation results were not as consistent across validation methods. For the mid-parent validation (FY only), some bias (0.85 and 0.91) and over-dispersion (0.63 and 0.52) were present in both the traditional and genomic evaluations even with adjusted phenotypes (Table 3). For the forward validation methods, with adjusted phenotypes and the LR method (Tables 4 and 5), bias was generally small for FY (− 0.09 to 0.33) but overall larger for BW (− 12.61 to 242.13). Large differences in the absolute values of bias are likely due to the scale of the phenotypes for the traits, making the comparison between traits more difficult. Representing these results as a proportion of the genetic standard deviation (SDa) for each trait shows that the bias for FY was more severe (from 6 to 23% of SDa) than for BW (up to 2% of SDa).

The $\mathrm{b}1$ coefficient ranged from 0.96 to 1.04 for FY and from 0.74 to 0.97 for BW, indicating small under- and over-dispersion of the estimates of breeding values for the forward validation methods.

In our study, we constructed $\mathbf{G}$ using base allele frequencies because these are preferable when available [27], since they ensure compatibility between $\mathbf{G}$ and ${\mathbf{A}}_{22}$. In addition, genotyping in this population covered many samples within the same family (i.e., around 5 fish per family in each year-class); therefore, many genotyped fish represented the variability within the family, and few represented the variability across families. This creates stratification, and the estimation of allele frequencies based on the current population can be relatively poor in such a scenario. Although base allele frequencies were used, dispersion bias was still observed when validating on future performances. To further explore the potential sources of biases in our evaluations, we could analyze different aspects of the population structure. For instance, although selection for FY is recent in this population, it has been selected for growth performance for five generations prior to the FY selection that started in 2014 [19, 21]. This prior selection could lead to selective genotyping of the animals with superior genetic merit for growth in the recent generations, which can be difficult to account for and may lead to biased predictions [40]. Another source of bias in the evaluations could be due to the different fixed effects combinations (results not shown) included in the model. Bermann et al. [38] showed that validation based on future performances (i.e., adjusted phenotypes) is more sensitive to model specification than that based on future GEBV (i.e., LR method).

As pointed out by Legarra and Reverter [41], even when prediction accuracy is higher, bias and inflation of breeding values could lead to less than optimal selection of animals. Although it may be less important for species with discrete generations, measures to mitigate these biases should be put in place, and an evaluation with a higher prediction accuracy and lower bias and inflation should be preferred.

### Consistency of evaluations

In addition to accuracy, bias, and inflation, the LR method provides a measure of consistency between evaluations. This consistency is based on the correlation of the breeding values of validation animals using the whole data with those using the partial datasets, and a higher correlation means that the partial data predicts well the whole data. In our study, ssGBLUP was more consistent compared to PBLUP for FY (0.79 vs 0.63) and for BW (0.68 to 0.52), as shown in Table 5. This result is expected because as the genomic evaluation is more accurate, the breeding values tend to change less when more data is added in subsequent evaluations.

### Weighted ssGBLUP (WssGBLUP)

Weighted ssGBLUP results for FY and BW are in Tables 6 and 7, respectively. Generally, using different weights for SNPs by WssGBLUP did not yield improvements in prediction accuracy for the studied traits, and prediction accuracy was similar to that with ssGBLUP. However, when CT was set to 1.125 there was a marginal increase in accuracy from 0.62 to 0.63, on iterations 4 and 5 for FY (Table 6). In spite of similar accuracies, there were differences in bias and in the inflation of breeding values. Overall, the use of weights resulted in an increase in bias, for instance, when CT was set to 1.125 for FY, $\mathrm{b}0$ ranged from − 0.44 to − 0.60 (iterations 1 to 5), whereas for BW, when CT was set to 1.25, $\mathrm{b}0$ ranged from 44.22 to 59 (iteration 1 to 5), both representing an increase in bias. Small changes in inflation could also be observed based on CT values and iterations of WssGBLUP for both traits. Although in some cases, accuracy was slightly increased or inflation slightly reduced, such marginal changes would not considerably improve the predictions. For polygenic traits, such as FY and BW in our study, weighting SNPs differently is not expected to increase prediction accuracy. However, for a trait such as resistance to BCWD in rainbow trout that is influenced by two or three major QTL it was shown that WssGBLUP and BayesB consistently generated higher prediction accuracy [9, 42]. Some studies have shown minor improvements in prediction accuracy with WssGBLUP when SNPs close to a major QTL are given bigger weights [43] or when selected sequence variants are added to the SNP panel [44].

**Table 6:** Results for WssGBLUP for fillet yield for different weighting strategies for the SNPs

Iteration	1.025^a^					1.125					1.25				
	1	2	3	4	5	1	2	3	4	5	1	2	3	4	5
Accuracy	0.62	0.62	0.62	0.62	0.62	0.62	0.62	0.62	0.63	0.63	0.62	0.62	0.62	0.62	0.62
b0	− 0.44	− 0.51	− 0.52	− 0.52	− 0.52	− 0.44	− 0.57	− 0.60	− 0.60	− 0.60	− 0.44	− 0.46	− 0.46	− 0.46	− 0.46
b1	1.07	1.03	1.03	1.03	1.03	1.07	1.00	0.99	0.99	0.99	1.07	1.06	1.06	1.06	1.06

^a^CT values tested, CT is a constant that represents the deviation from normality in the calculation of weights for the SNPs

b0: bias; b1: inflation

Validation is done with adjusted phenotypes

**Table 7:** Results for WssGBLUP for body weight for different weighting strategies for the SNPs

Iterations	1.025^a^					1.125					1.25				
	1	2	3	4	5	1	2	3	4	5	1	2	3	4	5
Accuracy	0.39	0.39	0.39	0.39	0.39	0.39	0.39	0.39	0.39	0.39	0.39	0.39	0.39	0.39	0.39
b0	44.22	45.34	45.36	45.36	45.36	44.22	49.57	50.24	50.45	50.53	44.22	54.29	57.77	58.92	59.00
b1	0.83	0.83	0.83	0.83	0.83	0.83	0.80	0.80	0.80	0.80	0.83	0.77	0.76	0.75	0.75

^a^CT values tested. CT is a constant that represents the deviation from normality in the calculation of weights for the SNP markers

b0: bias; b1: inflation

Validation are done with adjusted phenotype

### Genome-wide association study (GWAS)

**Fig. 1:**
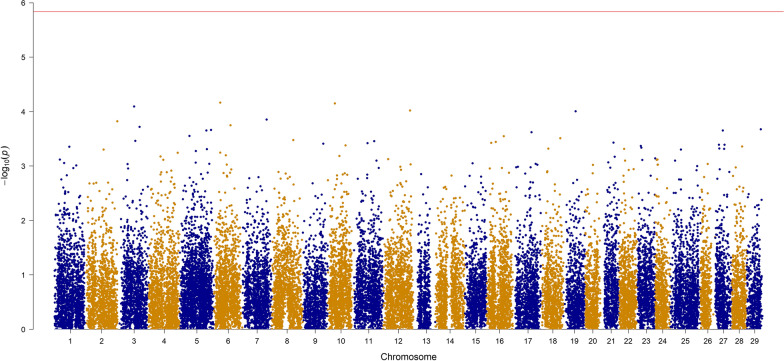
Manhattan plot for FY with − log10 p-values

**Fig. 2:**
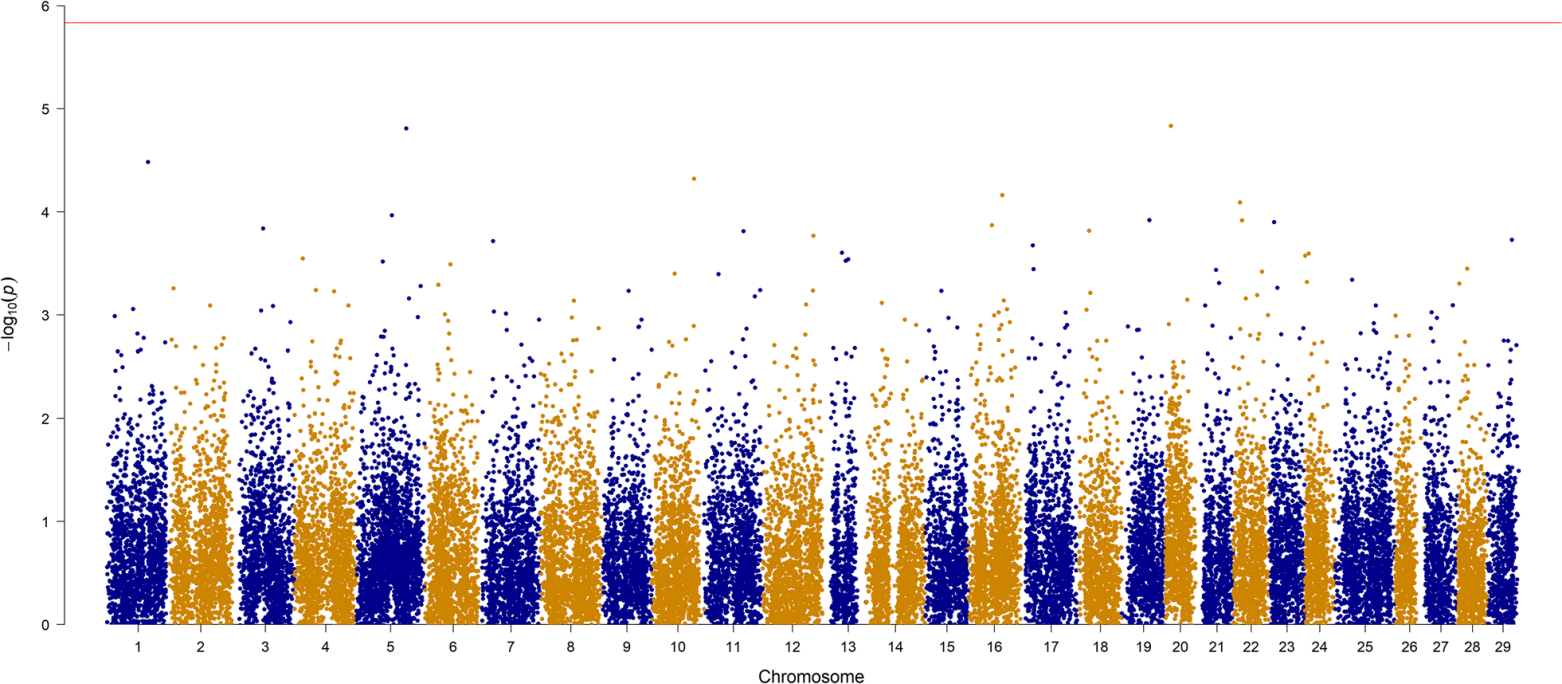
Manhattan plot for BW with − log10 p-values

**Fig. 3:**
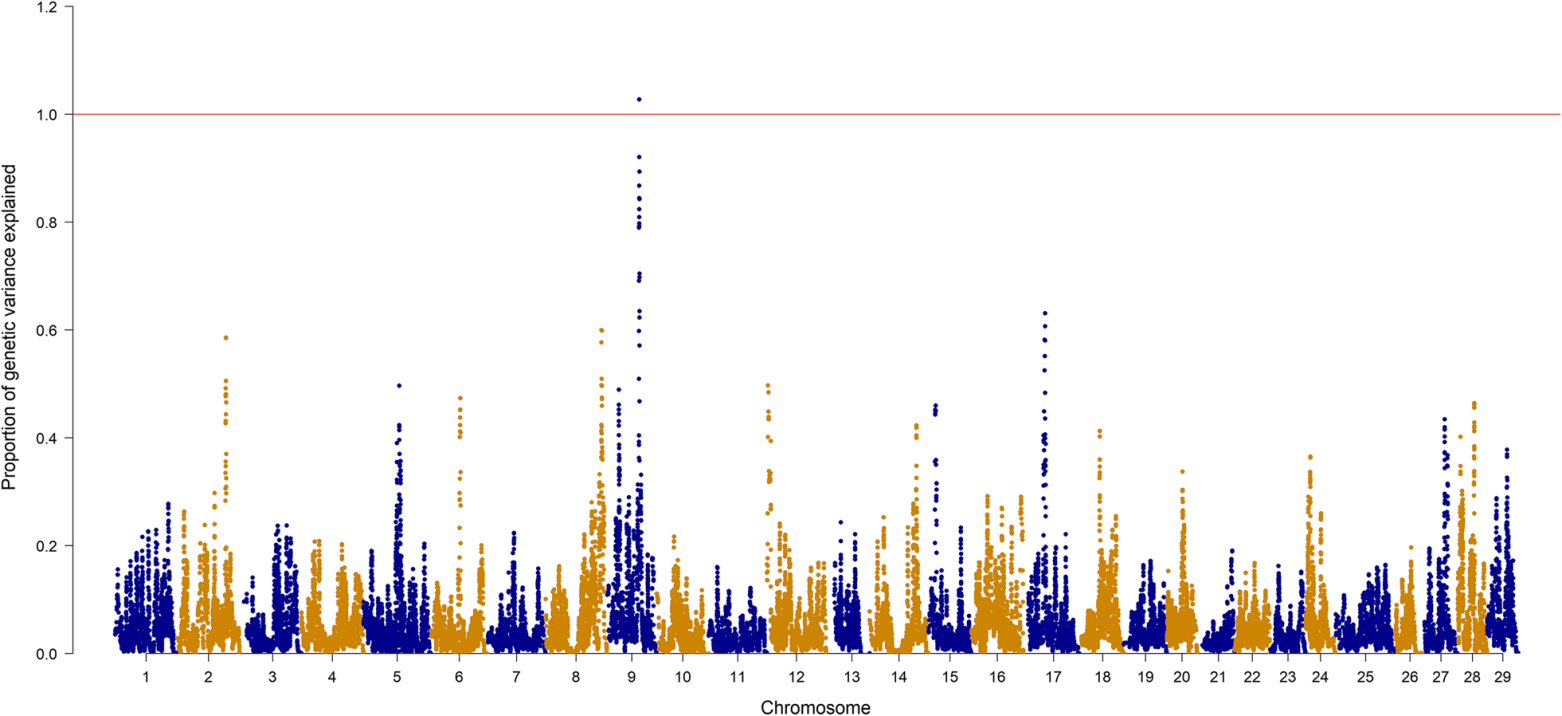
Manhattan plot for FY with the proportion of additive genetic variance explained by 20 adjacent SNPs

**Fig. 4:**
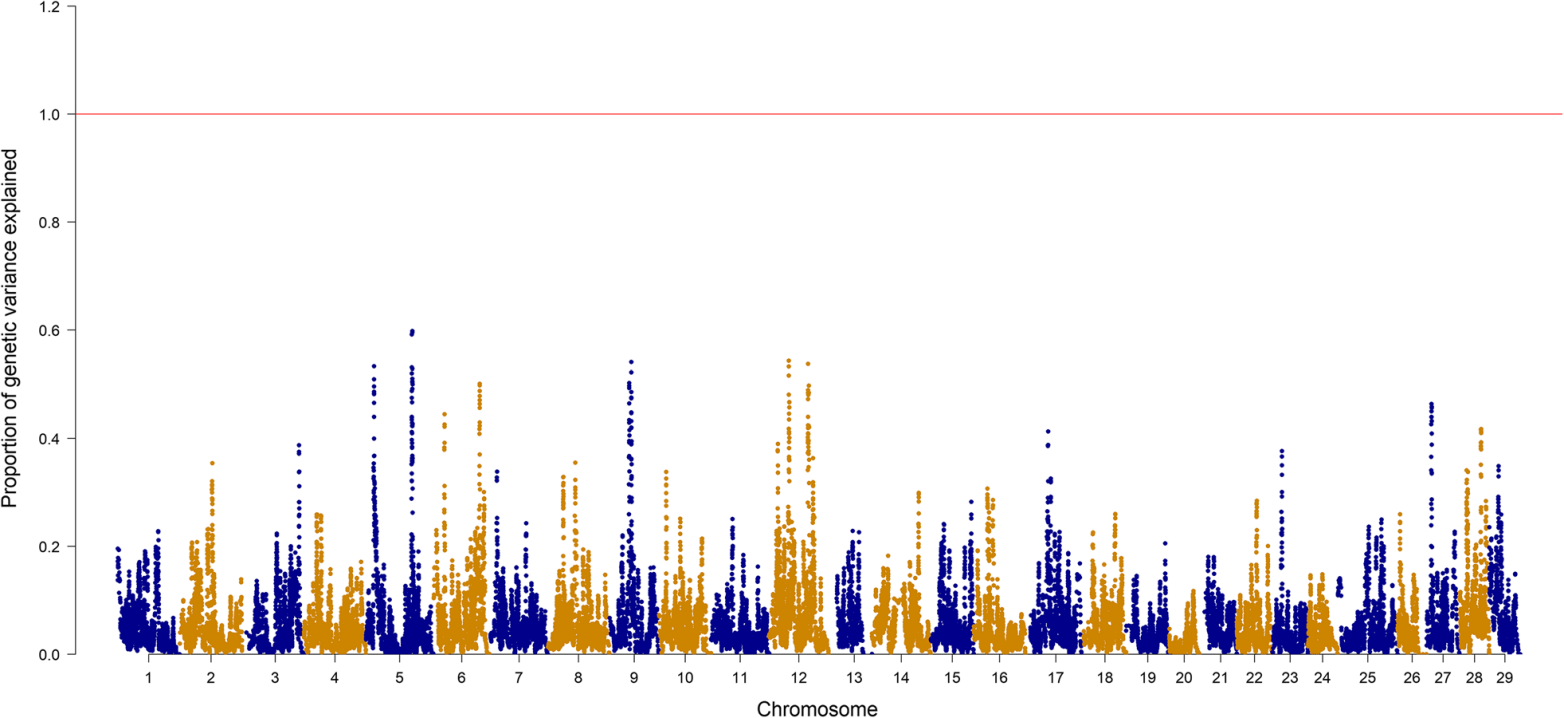
Manhattan plot for BW with the proportion of additive genetic variance explained by 20 adjacent SNPs

Figures 1 and 2 show the Manhattan plots for FY and BW with the p-values. Based on the p-values calculated from ssGBLUP and a Bonferroni threshold of 5.8, none of the SNPs were declared significantly associated with the traits. In addition, Figs. 3 and 4 show the proportion of additive genetic variance explained by windows of 20 adjacent SNPs. For FY, one window on trout chromosome Omy9 explained up to 1.02% of the genetic variance (Fig. 3), and for BW, three windows on Omy6 explained up to 0.6% of the genetic variance. Given that no SNP were declared significant based on the p-values and that the proportion of variance explained was small for both traits, the polygenic nature of both FY and BW is confirmed.

Other studies have investigated the genetic architecture of FY in rainbow trout. For example, Gonzalez-Pena et al. [19], using a single-trait model, found similar results, with windows explaining up to 1.5% of variance for FY on Omy9 and one window on Omy5 explaining 0.95% of the variance of BW. As in our study, the authors concluded that both traits are polygenic and can benefit from genomic selection using all available markers. However, a more recent study by Salem et al. [45] found two windows of 50 SNPs explaining 12.71% and 10.49% of the genetic variance for FY on Omy 14 and 16, respectively, using the same sample as in Gonzalez-Pena et al. [19] but a different 50K SNP array to genotype the fish. Their array was developed using SNPs that had differential allelic frequencies between high and low growth families from the same studied population. The SNP array that was used by Gonzalez-Pena et al. [19] and in our study was based on SNPs that were shown to be polymorphic in a wide range of rainbow trout populations [20]. In addition, we specifically examined the QTL regions reported in Salem et al. [45] and found that they are equally represented compared to the rest of the genome in the SNP array used in our study but are highly enriched with SNPs from the array used by Salem et al. [45] (data not shown). Therefore, this discrepancy in the reported QTL may be caused by allelic ascertainment bias and differential enrichment for certain genome loci between the SNP arrays used in each study.

## Conclusions

The low but positive genetic correlation between fillet yield and body weight indicates that some improvement in fillet yield may be achieved through indirect selection for body weight. Genomic information increases the prediction accuracy of breeding values and is an important tool to accelerate genetic progress for fillet yield and growth in the current rainbow trout population. No major SNPs were found to be significantly associated with the studied traits, which suggests that using all the SNPs available in the panel for genomic evaluations is a better strategy. Weighting SNPs differently provides only a marginal increase in prediction accuracy compared to the use of the unweighted single-step model. This indicates the existence of many loci with small effects on these traits in the genome. Past selection for growth rate, selective genotyping, and the relatively small number of genotyped animals in the current populations are possible sources of bias in the evaluation for fillet yield and body weight.

## Data Availability

The datasets used and/or analyzed during the current study are available from the corresponding author on reasonable request.
